# Supplementary material for: Time-series transcriptome analysis identified differentially expressed genes in broiler chicken infected with mixed Eimeria species
Source: Front Genet. 2022 Aug 8;13:886781. doi: 10.3389/fgene.2022.886781 (PMC9393255; doi:10.3389/fgene.2022.886781)
Supplement: Supplementary file 2 [file DataSheet1.ZIP › 7dpi_GO.Gsea.1625106495923/GOBP_COLLATERAL_SPROUTING.html]

Details for gene set GOBP\_COLLATERAL\_SPROUTING[GSEA]

|  || Dataset | TMM\_7dpi\_gct\_format\_7dpi\_gct\_format.Class\_4dpi.cls #PC\_versus\_NC.Class\_4dpi.cls #PC\_versus\_NC\_repos |
| Phenotype | Class\_4dpi.cls#PC\_versus\_NC\_repos |
| Upregulated in class | 0 |
| GeneSet | GOBP\_COLLATERAL\_SPROUTING |
| Enrichment Score (ES) | -0.7034181 |
| Normalized Enrichment Score (NES) | -1.824601 |
| Nominal p-value | 7.698229E-4 |
| FDR q-value | 0.11319331 |
| FWER p-Value | 0.8916 |
Table: GSEA Results Summary

  

Fig 1: Enrichment plot: GOBP\_COLLATERAL\_SPROUTING      
 Profile of the Running ES Score & Positions of GeneSet Members on the Rank Ordered List

  

| SYMBOL | TITLE | RANK IN GENE LIST | RANK METRIC SCORE | RUNNING ES | CORE ENRICHMENT || 1 | RND2 | na | 1081 | 0.492 | -0.0481 | No |
| 2 | SPP1 | na | 2272 | 0.226 | -0.1280 | No |
| 3 | APP | na | 4058 | -0.002 | -0.2766 | No |
| 4 | EPHA7 | na | 4298 | -0.030 | -0.2940 | No |
| 5 | IST1 | na | 5951 | -0.196 | -0.4149 | No |
| 6 | ABL1 | na | 6549 | -0.259 | -0.4426 | No |
| 7 | PTPRS | na | 7463 | -0.357 | -0.4882 | No |
| 8 | LRP1 | na | 9005 | -0.550 | -0.5697 | No |
| 9 | COBL | na | 10610 | -0.835 | -0.6321 | Yes |
| 10 | DVL1 | na | 10694 | -0.853 | -0.5661 | Yes |
| 11 | NIN | na | 10931 | -0.916 | -0.5076 | Yes |
| 12 | SEMA4D | na | 11524 | -1.127 | -0.4607 | Yes |
| 13 | LPAR3 | na | 11920 | -1.562 | -0.3602 | Yes |
| 14 | FSTL4 | na | 11989 | -1.962 | -0.1983 | Yes |
| 15 | FGF13 | na | 12005 | -2.341 | 0.0004 | Yes |
Table: GSEA details [plain text format]

  

Fig 2: GOBP\_COLLATERAL\_SPROUTING      
 Blue-Pink O' Gram in the Space of the Analyzed GeneSet

  

Fig 3: GOBP\_COLLATERAL\_SPROUTING: Random ES distribution      
 Gene set null distribution of ES for **GOBP\_COLLATERAL\_SPROUTING**
